# Multi-locus investigation of Anopheles-mediated selective pressure on Plasmodium falciparum in Africa

**DOI:** 10.21203/rs.3.rs-5040478/v1

**Published:** 2024-10-30

**Authors:** Isuru Gunarathna, Joseph D. Spear, Tamar E. Carter

**Affiliations:** Baylor University; Baylor University; Baylor University

**Keywords:** vector-parasite interactions, vector-mediated selection, genomics, coevolution

## Abstract

**Background:**

The high burden of malaria in Africa is largely due to the presence of competent and adapted *Anopheles* vector species. With invasive *Anopheles stephensi* implicated in malaria outbreaks in Africa, understanding the genomic basis of vector-parasite compatibility is essential for assessing the risk of future outbreaks due to this mosquito. Vector compatibility with *P. falciparum* arises from ancient coevolution and involves genes like *Pfs47* in *P. falciparum* and P47Rec in *Anopheles*. Questions remain about whether sub-continental vector variation is a selective pressure on current *Plasmodium* populations or not.

**Methods:**

We analyzed the genetic diversity in parasite-vector interaction genes in *P. falciparum* and *An. gambiae* from 9 and 15 countries in Africa, respectively. Specifically, we looked for evidence of malaria vector-mediated selection within three *P. falciparum* genes *(Pfs47, Pfs16, Pfs37)* and conducted association analyses with occurrence probabilities of prominent malaria vectors (VOP).

**Results:**

Higher protein haplotype diversities of Pfs47 and Pfs16 were associated with the probability of occurrence of *An. arabiensis* and *An. funestus* together. Only *Pfs16* carried a signature of positive selection consistently (average Tajima’s D = −2.96) which was associated with the probability of occurrence of *An. funestus*. These findings support vector-mediated selection based on vector species diversity may be occurring within Africa. We also employed phylogenetic analyses of *An. gambiae* interaction genes (*P47Rec*, *APN1*, *HPX15*) to identify significant subspecies diversity as a prerequisite to vector-population-mediated selection. *An. gambiae* HPX15 revealed significant sub-species differentiation (multiple branches bootstrap >70) compared to absence of variation in P47Rec, suggesting further investigation into sub-species mediated selection based on HPX15 is needed. Finally, we observed five amino acid changes at P47Rec in invasive *An. stephensi* compared to dominant African *Anopheles*species, calling for further investigation of the impact these distinct P47Rec variants would have on local African *P. falciparum* Pfs47 diversity.

**Conclusion:**

Overall, these findings support the notion that vector variation within Africa could influence *P. falciparum*diversity and lay a genomic framework for future investigation of invasive *An. stephensi’s* impact on African malaria.

## 1. BACKGROUND

In 2022, over 249 million cases of malaria were reported, with the majority of cases located in Africa (1). Efforts to control this disease, which impacts half of the world’s population, have reached a critical point in the last few years following the ten percent increase in cases observed in 2020 (2). Most of these cases are due to the unicellular eukaryotic parasite *Plasmodium falciparum* adapted to spread through *Anopheles gambiae sl.* mosquitos present throughout most of the African continent (3). Most recently, *Anopheles stephensi*, common to South Asia and the Middle East, has invaded the Horn of Africa and several other African countries further exasperating malaria control (4–6). The vectorial competence, breeding habitats and behaviors vary even among the members of the native *An. gambiae sensu lato* complex (7). With the invasion of *An. stephensi* now contributing to increased complexity of the already diverse vector species composition in Africa, it is important to determine the new transmission dynamics in *An. stephensi* invaded areas.

Interactions between *Plasmodium* and the mosquito midgut serve as the critical gateway for malaria transmission. The parasite invasion of the mosquito midgut requires an interaction between both parasite and mosquito proteins (8). Previous studies have shown that the level of compatibility of interacting proteins between malaria vector species and parasite species varies depending on the haplotypes of the genes coding for these proteins, especially during the midgut invasion (9). Many of the *Anopheles* and *Plasmodium* genes responsible for these interactions are currently being studied for their potential use in development of transmission blocking vaccines (10, 11).

In addition to within-species genetic diversity, the haplotype diversity of genes involved in interactions with malaria parasites across different vector species may influence the genetic diversity of key genes that mediate vector-parasite interactions in *Plasmodium falciparum*. Different mosquito species, such as *Anopheles gambiae* and *Anopheles funestus*, have varying ecological niches, behaviors, and interactions with the malaria parasite, which can lead to differential selective pressures on the parasite’s genes (12). For instance, variations in the vector’s immune response, feeding habits, and geographical distribution can drive genetic diversity in the parasite as it adapts to survive and thrive in different vector environments (13). Consequently, regions with diverse vector species compositions are likely to exhibit higher nucleotide and haplotype diversity in *P. falciparum* genes associated with vector interactions, reflecting the parasite’s adaptation to a range of vector-related selective pressures.

A well-studied example for parasite-vector interaction genes is the Pfs47-P47Rec complex in *P. falciparum* and *An. gambiae* (11, 14, 15). The mosquito midgut protein P47Rec and parasite protein Pfs47 work as a receptor-ligand pair during the *Plasmodium* invasion by playing a role in the immune evasion of parasites to make the parasite “undetectable” to the mosquito immune system. Silencing *P47Rec* expression has reduced the infection of *P. falciparum* in *An. gambiae* mosquitos (16).

With this “lock-and-key” type mechanism, the ability of *P. falciparum* strains to invade the *Anopheles* midgut cells is dependent on the correct matching of Pfs47 surface protein haplotype (“the key”) with the *Anopheles* midgut receptor P47Rec (“the lock”) (16). Previous functional studies demonstrated that replacing Pfs47 haplotype in African *P. falciparum* with a different haplotype from another continent is suficient to change the compatibility between the vector and parasite (9). Later studies have shown that *Pfs47* is important for the adaptation of *Plasmodium falciparum* to different malaria vectors (17). This vector-mediated selective pressure at the continental level in *Pfs47* resulted in significant population structure between different continents, particularly in domain 2 of the protein (14, 18, 19). Sub-continental selective pressure on the *Pfs47* has been observed in previous studies in Nigeria, Brazil, and Malaysia (19). Still, significant knowledge gaps remain about the level of vector-mediated selective pressure on *Pfs47* at a sub-continental level in Africa. This is important to evaluate given the multiple *Anopheles* vector species that exist sympatrically across Africa, some quite divergent from one another (eg. the *An. gambiae* complex vs. *An. funestus*) (20).

In addition to the Pfs47-P47Rec system, there are several other protein coding genes being studied as transmission blocking vaccine (TBV) targets based on their role in parasite-vector compatibility. Therefore, like *Pfs47* and *P47Rec*, these parasite and vector genes may also involve vector-mediated selection (10, 15). In our study we selected two more genes from *P. falciparum* and another two genes from *An. gambiae* which are important for vector-parasite interactions. In *Plasmodium* parasites, *Pfs16* (PF3D7_0406200) and *Pfs37* (PF3D7_1204400) have been recognized to be important for vector-parasite interactions because of their significant upregulated expression in the sexual stages and interactions with mosquito midgut proteins (15). Knocking out *Pfs16* or *Pfs37* has shown a reduction in the number of oocysts generated in the mosquito midgut during the parasite invasion (15, 21). Initially *Pfs16* was suspected to be required for optimal production of sexual stage parasites (22).

At the other end of the vector-parasite interaction equation, *Anopheles* midgut proteins AnAPN1 and HPX15 have been recognized for their importance in vector-parasite interactions and for their significant impact on the survival of the parasite. HPX15 is an immune-related protein with pattern-recognition molecules and previous studies indicate that it promotes malaria transmission (10). Specifically, HPX15 plays a role in the preservation of the functionality of stored sperm and long-term fertility in *An. gambiae* (23). In *An. stephensi* mosquitos, RNA interference-mediated silencing of midgut AsHPX15 gene has drastically reduced the number of developing *P. berghei* oocysts (24). Alanyl aminopeptidase N (AnAPN1) is a protein that can elicit transmission-blocking antibodies, which is believed to be highly conserved among *Anopheles* vectors (25), though not thoroughly investigated across Africa. The effectiveness of antibodies targeting AnAPN1 against *P. falciparum* and *P. vivax* across distantly related *Anopheles* species is well studied (26).

While there is strong support of ancient vector-mediated selection on *Plasmodium* by continentally structured *Anopheles* species, questions remain about the potential for ongoing vector-mediated selection within a continental region. The goal of this study was to evaluate the potential for vector-mediated selection on parasite populations within African countries by examining the patterns of diversity in vector-parasite interacting genes. To better understand the sub-continental dynamics of vector-mediated selection, we aim to investigate the genetic diversity and selection signals in *P. falciparum* interaction genes, along with the vector species composition and subspecies variation in *An. gambiae* interaction genes. This study will specifically address the implications of *Anopheles stephensi* invasion by providing insights into the nature of these interactions on a finer scale.

## 2. METHODS

All the command line-based programs were run on the operating System: Rocky Linux 8.8 (Green Obsidian), Architecture: x86–64. The rest of the steps were carried out on a Windows (version 11 Education, 64-bit operating system) PC.

### 2.1 Data selection

We needed gene sequences from *P. falciparum* and *An. gambiae* from the malaria-endemic regions for this study. Therefore, Ag1000 and PF6K datasets shared by MalariaGen data-sharing network were used (27). In this study, abiding to the Ag1000 terms of use, we did not use more than 10% of the genome data and we did not report any genome wide statistics. When downloading genomes of vectors and parasites, sample sets were selected as separate populations, where at most 25 samples were collected from the same country in the same year. This number of samples (n = 25) was selected to hold the balance between representation of mosquitos from a particular region and computational power required to perform the analysis. Another reason to select the same number (or close to 25) of samples for every population was to avoid the increment of number of haplotypes due to the large number of samples. The *P. falciparum* samples with high probability of multiple infections (F_ws_ < = 0.95) were removed from the dataset using the F_ws_ values calculated by the authors of the Pf6K dataset (27). Abiding to above criteria, 418 *P. falciparum* genomes and 625 *An. gambiae* genomes were downloaded for 9 and 15 African countries, respectively. The ENA accession identifiers with country and year data are saved in Supplementary table 1 and 2 CSV files.

### 2.2 Quality control

The downloaded genome sequences were subjected to a quality control process using the FastQC v0.12.1program (28). First, each sample was screened for quality by examining the report generated by FastQC program. We specifically looked at the basic statistics, per base sequence quality, per base N content, and sequence length distribution to find anomalies in sequences. Trimmomatic v 0.39 program was used in trimming the low-quality regions of the reads in the dataset (29).

### 2.3 Sequence alignment to the reference genome.

The sequences of the samples were aligned to the *An. gambiae*, and *P. falciparum* genomes downloaded from Vectorbase (https://vectorbase.org/vectorbase/app) and Plasmodb (https://plasmodb.org/plasmo/app) databases. Sequences were aligned using the Bowtie2 (version 2.5.1) program on a Linux platform to create separate “.bam” files for each parasite and vector sample (30).

### 2.4 Variant calling and gene sequence handling.

The variants were called from the aligned “.bam” files using the mpileup option in BCFtools (version 1.17) program (31). Variants were normalized and filtered to get the highest quality variant call respective to the reference genomes. Only the biallelic variants were filtered out with Phred-scaled quality scores greater than 30, read depth greater than 10 and frequencies higher than 1%. The sequences of the interested genomic regions were extracted from the VCF files using Samtools program (version 1.18) and consensus option in BCFtools program. Extracted sequences were saved in FASTA format for the downstream analysis. VCF files of the mosquito genomes were phased using Segmented HAPlotype Estimation & Imputation Tool (shapeit2 – version 2.r904) to address the ploidy level. The gene sequences were aligned using Clustal and Muscle programs (32, 33).

### 2.5 Diversity statistics calculation

For all the genes studied here, for each population in both vectors and parasites, we calculated the Tajima’s D values, F_st_ values, nucleotide diversity, and haplotype diversity using the Pegas package (version 1.3) in R statistics (version 4.3.2) (34). Additionally, Genome-wide F_st_ and Tajima’s D values were calculated for comparisons. Values were recorded in tables for further analyses and visualized using the ggplot2 package in R statistics. Tajima’s D values were calculated according to the method described by F. Tajima in 1989 (35). Tajima’s D values were recorded with the corresponding beta p-values for each population to facilitate selecting statistically significant signals of selection. Nucleotide diversity and haplotype diversities for each population pair were calculated as described in M. Nei in 1987 and M. Nei and F. Tajima in 1981 respectively (36, 37). Pairwise F_st_ for each population pair was calculated using “gene.dist()” function in hierfstat (version 0.5.11) package in R statistics using the method described by Weir and Cockerham in 1984 (38). Genome-wide Tajima’s D value and Fst values were calculated using VCFtools (version 0.1.16).

### 2.6 Investigation of relationships between vector occurrence probabilities and parasite gene haplotype diversities

In this step we investigated the relationships between amino acid haplotype diversities of the genes important for interacting with the vectors in parasites and the probability of occurrence of prominent malaria vectors in Africa. We downloaded predicted vector occurrence probabilities (both 2010 and 2017 predictions) for the locations of parasite samples were collected from Malaria Atlas Project (39, 40). Correlation analyses were performed between the amino acid haplotype diversities of parasite genes and vector occurrence probabilities (VOP) using “cor.test” function on R statistics platform. For the vector species which had a statistically significant relationship with the haplotype diversity of parasite genes, regression models were fitted using “lm” function to examine the interaction between vector species occurrence probabilities on haplotype diversities of parasite genes. Furthermore, to reduce the uncertainty and noise inherent in vector occurrence probability data that were used in linear regressions, the probabilities were converted to a binomial variable of presence or absence of the vector species. We employed three distinct cutoff values for occurrence probabilities (0.5, 0.75, and 0.95) to assess vector presence, as a definitive rationale for selecting a single threshold was not available. Regression models were fitted to predict the amino acid haplotype diversities of the parasite genes against binomial vector occurrence of significantly correlated vector species as the predictor variables. Results of all the regression models were tabulated in an Excel sheet (Supplementary data sheet 2 - Combined sheet). In addition to the regression analyses, we categorized and visualized the haplotype diversities and Tajima’s D values of parasite populations based on the presence or absence of different combinations of vector species significantly associated with parasite gene haplotype diversity (Supplementary Figs. 4 and 5). This approach was specifically designed to identify patterns in haplotype diversities across various vector combinations that were not captured by the linear regression models.

### 2.7 *An. stephensi* mosquito collection and P47Rec sequence extraction

Samples were collected from September to November of 2018 in northeastern and eastern Ethiopian cities Semera and Kebridehar as previously detailed in (41, 42) and (6). Mosquitoes (n = 7) were collected using Centers for Disease Control and Prevention light traps and pyrethrum spray collection in houses, and larvae and pupae were sampled using the WHO dipping approach. DNA was extracted from the (head and thorax?) using the Qiagen DNeasy kit. Once the DNA was extracted, the *P47Rec* ortholog in *An. stephensi* was amplified using two primer pairs. The first pair (forward – 5’-TGGCAAATGACTAACGTGGA-3’, reverse – 5’-GTGTTGCCAGTTCGCTGTAA-3’) amplified the second and third exons while the second pair (forward – 5-GTGAGCAGCTGTACGTTGGA-3’, reverse – 5-AAAACGGAAGGCATGTCATAA-3’) amplified the fourth exon. Sequences were aligned using the MUSCLE program and a maximum likelihood tree was generated using the RAxML version 2.0 program (43).

## 3. RESULTS

### 3.1 Population structure and polymorphism in *Plasmodium falciparum* genes

**Figure 1:**
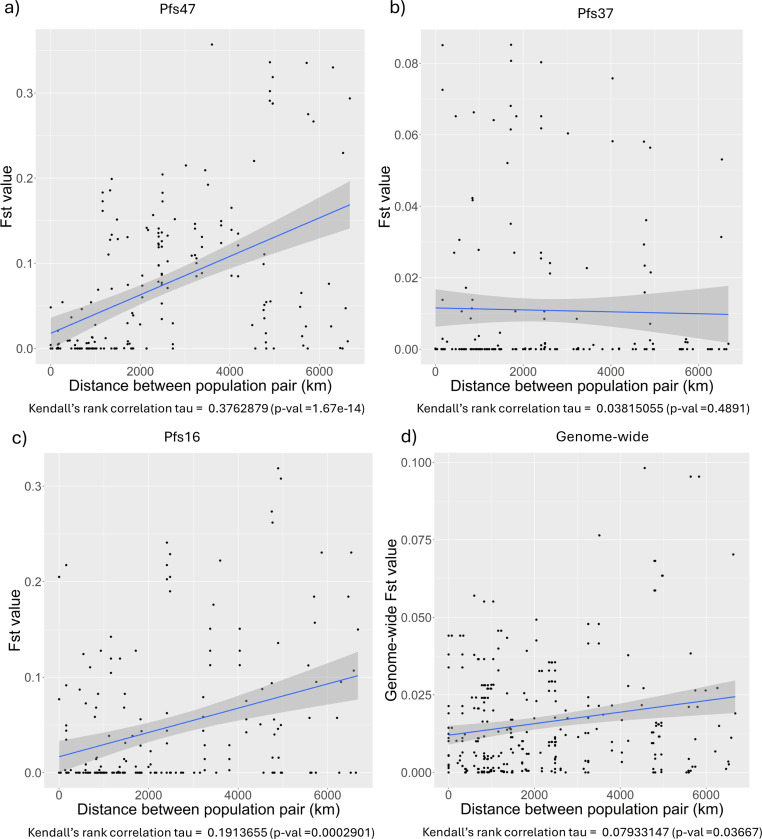
Correlation between pairwise F_st_ and geographic distance between populations of *P. falciparum* genes and genomes in Africa. Blue line represents the regression line fitted for the data points. The correlation coeficient was calculated for each gene and noted down below each scatterplot. Panels a through c show the correlation between pairwise F_st_ values and geographic distances for the three genes Pfs47, Pfs37 and Pfs16 respectively and panel d is the genome-wide correlation analysis.

To investigate the population structure and its association with geographic distribution of *P. falciparum* genes, we measured the pairwise F_st_ between each population pair and tested for correlation with geographic distance between populations (see Fig. 1). For Pfs47, pairwise F_st_ values ranged between 0 and 0.3569732. The highest values were observed in the Malawi population against other populations. Malawi was also relatively (not statistically) isolated from other Central African countries including the Democratic Republic of Congo and Cameroon which were relatively (not statistically) isolated from West African populations (see supplementary Fig. 1). In Pfs47, a statistically significant but weak correlation was observed between pairwise F_st_ values and the geographic distances among the *P. falciparum* populations (see Fig. 1).

In parallel to Pfs47, Pfs16 also showed a statistically significant, but weak correlation between pairwise F_st_ values and the geographic distances among the *P. falciparum* populations (Kendall’s rank correlation tau = 0.1913655, p-value = 0.0002901). Again, Malawi had the highest pairwise F_st_ values, but the isolation patterns were different between Pfs47 and Pfs16 (see supplementary Fig. 1). Both Pfs37 and genome-wide pairwise F_st_ values did not show statistically significant correlations with the distance between populations (Fig. 1).

**Figure 2:**
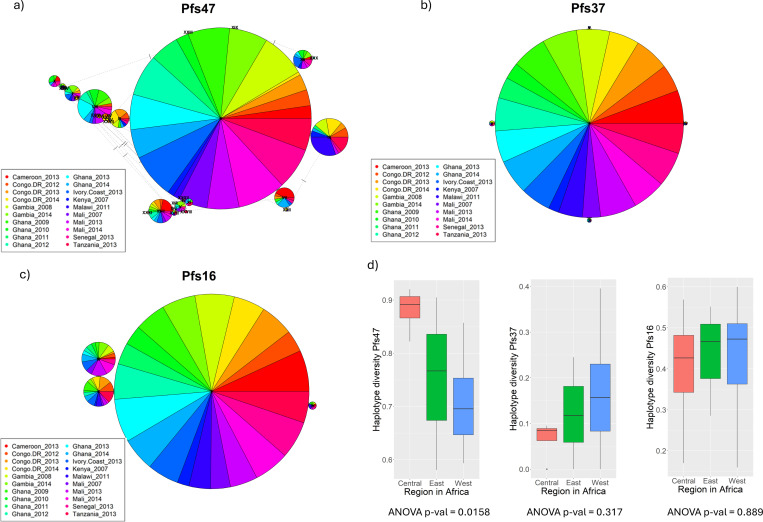
Haplotype networks (nucleotide) of the *P. falciparum* genes and boxplots with the levels of haplotype diversity (nucleotide) among three main regions of Africa (East, Central and West). Panels a through c, are haplotype networks of Pfs47, Pfs37 and Pfs16 respectively. Panel d) box plot of haplotype diversity levels among three major regions of Africa. ANOVA p-value was denoted under each boxplot of haplotype diversity levels in three main regions of Africa.

**Figure 3:**
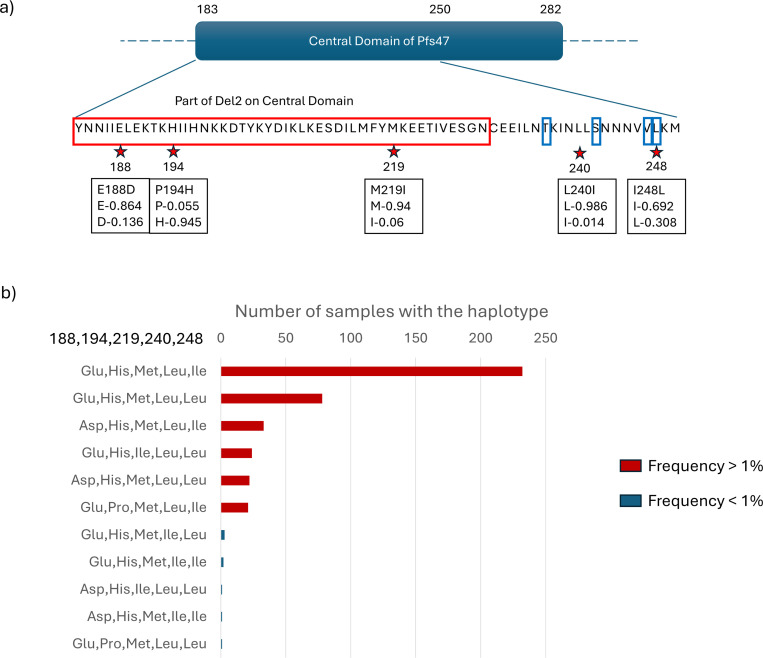
Non-synonymous mutations of Pfs47 domain 2 and their frequencies. a) the locations of the mutations on domain 2 of Pfs47 protein. The red stars denote the polymorphic sites within the region (183^rd^ aa to 250^th^ aa) and red box denotes Del2 region which is selected as antigen for TBV. The blue boxes show the previously identified mutations important for vector parasite interaction. Notation within the black boxes shows the mutation in the first line and frequency of each allele in second and third lines b) the bar plot shows the haplotypes in the 418 protein sequences of Pfs47 central domain (D2) with their frequencies collected in African countries.

Among *P. falciparum* in African countries we studied, the number of haplotypes for Pfs47, Pfs37, and Pfs16 were 32, 5 and 4 respectively (Fig. 2). We observed three polymorphic sites within the Del2 region in the central domain of Pfs47 (D2) which has been selected as a candidate antigen, which generates antibodies that block transmission (see Fig. 3) (11). Secondly, we observed two amino acid polymorphisms between the two cysteines in Pfs47-D2, the region known to be important for mosquito infectivity (see Fig. 3). To understand the geographical distribution of haplotypes in parasite populations we generated haplotype networks for the three parasite genes. In all three haplotype networks, especially the Pfs47 central domain (D2) haplotype network, there was roughly equal representation from all the populations indicating the presence of each haplotype in many parts of the continent (see Fig. 2 and Supplementary Fig. 2, 5). In Pfs47, haplotype II had the highest number of samples and represented most of the populations in dataset. To find out which parts of the African continent harbor the highest number of parasite gene haplotypes, we divided the continent into three regions (East, Central and West) and measured the haplotype diversity. Haplotype diversity level of Pfs47 was significantly higher in Central African countries (ANOVA F_(2, 17)_ = 5.344 p-value = 0.0158, Tukey’s HSD test p-values of West- Central and East-Central were 0.0119208 and 0.1825349 respectively, see Fig. 2 panel d). Compared to Pfs47, both Pfs37 and Pfs16 had haplotype diversity evenly distributed across the continent (see Fig. 2, d boxplot haplotype diversity means for Pfs37 and Pfs16). In Pfs47 there were 9 SNPs (Single Nucleotide Polymorphisms) and 5 of them had frequencies higher than 10% in the sample set we analyzed. Out of the nine SNPs, eight were non-synonymous mutations. There was a single SNP in Pfs16 coding sequence and an indel expanding from the 423rd base pair to the 428th base pair creating amino acid changes I85L, D140- and K141-. All these variations had a frequency higher than 10%.

### 3.2 Signals of selection in parasite genes

One of the goals of this study was to see whether the parasite interacting genes are evolving under positive selection in any of the African populations investigated here. Therefore, we calculated the Tajima’s D values for each parasite gene for each population and compared it with the average Tajima’s D values of the entire genome (−0.783259075 ) for the samples analyzed in this study. Tajima’s D values were calculated for each population and tabulated (see Supplementary data sheet 1). Among parasite populations, the average Tajima’s D over the entire genome varied between − 1.320805 and − 0.2383106. For each population, Pfs47 and Pfs37 did not show any statistically significant signals of non-neutral evolution (averaged Tajima’s D values − 0.420956776 and − 1.167216138 respectively, beta p-values > 0.05). However, Pfs16 had significantly higher negative Tajima’s D values in all the individual populations (average Tajima’s D value = −2.960073673, beta p-values < 0.05) indicating that it is evolving non-neutrally in many parts of the continent. Since the Pfs16 Tajima’s D values were significantly negative compared to the genome-wide Tajima’s D value this could be an indication of positive selection at this locus.

### 3.3 Population structure in *An. gambiae* genes

**Figure 4:**
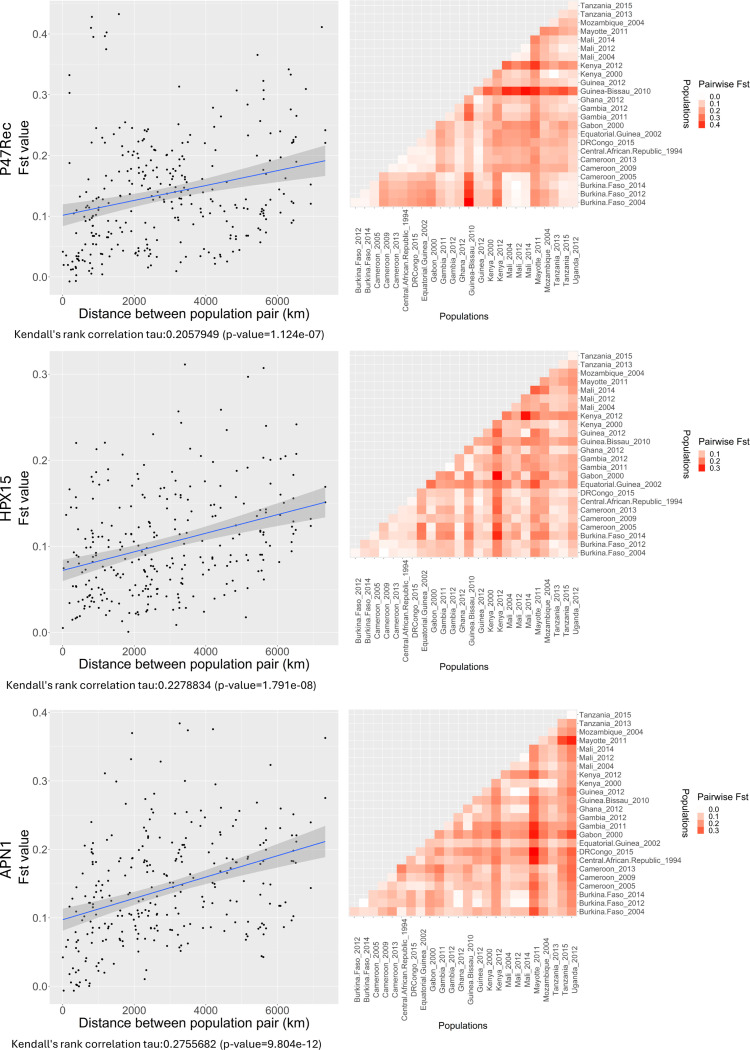
The scatter plots of *An. gambiae* genes pairwise F_st_ against geographic distance between populations. The heat maps represent the pairwise F_st_ values for each pair of populations.

To investigate the potential of influence of vector-parasite interactions on the population structure of parasites *Plasmodium*, we analyzed the population structure of genes (nucleotide sequences) coding for proteins known to be important for the survival of the parasite in the *An. gambiae* in several African countries. We measured the pairwise F_st_ between each selected *An. gambiae* population pair and tested for correlation with the geographic distance between populations (see Fig. 4). The F_st_ values varied between 0 and 0.4327906, 0.3112532, 0.3841258 for P47Rec, HPX15 and APN1 respectively. For the P47Rec gene, Guinea-Bissau and Kenya (2012) populations showed the highest level of isolation from other regions while Central African populations had a trend of being differentiated from the other populations. In HPX15, the Kenya (2012) population was again the most isolated population compared to the rest of the populations followed by Mayotte and Mozambique populations. However, in APN1 Mayotte population had the highest level of isolation followed by the Southeast African populations in Uganda, Tanzania, and Mozambique. Kenya (2012) was relatively (not statistically) different from the rest of the populations. All three genes studied here showed statistically significant but weak correlations between the pairwise F_st_ values and geographic distance among populations (see Fig. 4).

The number of SNPs that cause changes in amino acids were tabulated for each exon of the three genes (see Table 1). No non-synonymous mutations were detected in the P47Rec. However, 32 and 72 nonsynonymous mutations were detected in the HPX15 and APN1, respectively. Given the amino acid variation in these two genes, we were interested in the phylogenetic relationship among the mosquitoes. Phylogenetic analysis revealed support for the presence of distinct clades within *An. gambiae* for HPX15 (bootstrap values > 70) but not in APN1 (Supplementary Figs. 6 and 7).

### 3.4 Relationships between vector occurrence probabilities and parasite gene haplotype diversity

We conducted individual association analysis of the *P. falciparum* diversity statistics and occurrence probabilities of eight commonly occurring malaria vector species in Africa. Only Pfs47 and Pfs16 amino acid haplotype diversities had significant associations (Kendall’s rank correlation p-value < 0.05) with predicted occurrence probabilities of *An. arabiensis*, *An. funestus* and *An. moucheti* (see Table 2). Regression models were fitted to explain the amino acid haplotype diversities of Pfs47 and Pfs16 using the vector occurrence probabilities (VOP) of the above mentioned three vector species as continuous and binomial variables separately (Supplementary data sheet 2- Separate and Combined sheets). Models fitted with VOPs as continuous variables had significant overall p-values (< 0.05) and adjusted R-squared values greater than 0.48 but estimates for the each VOPs were close to zero. In the regression models fitted with VOPs as binomial variables, irrespective of the cutoff value (to determine the presence or absence of a vector species from occurrence probabilities), Pfs16 amino acid haplotype diversity was associated with *An. arabiensis* with a significant (< 0.05) p-value and an estimate close to zero while Pfs47 was associated with *An. arabiensis* only under a cutoff value of 0.5. Apart from the linear regression analyses between parasite gene haplotype diversities and vector occurrence probabilities, we visualized the haplotype diversities observed under occurrence of different combinations of three vector species (*An. arabiensis*, *funestus* and *moucheti* – occurrence was determined by multiple threshold values as previously mentioned) that were significantly associated with higher haplotype diversities (Supplementary Fig. 4). We observed in most cases irrespective of the threshold level used to define the probable presence or absence of the vector species based on their predicted occurrence probability, the combination of *An. arabiensis* and *An. funestus* was associated with higher levels of amino acid haplotype diversity in Pfs47 and Pfs16 (Supplementary data sheet 2 - ANOVA sheet).

We investigated the relationship between Tajima’s D values of *Pfs16* and vector occurrences because it was the only gene that had signals of significant positive selection. We observed significant estimates for *An. funestus* VOP in both regression models fitted to explain *Pfs16* Tajima’s D using vector occurrence probabilities as continuous variables and binomial variables (see Supplementary data sheet 2). It is important to mention that *Pfs16* Tajima’s D values did not have a significant association with the region of Africa (Central, East or West) as observed in the results of the one-way ANOVA table (*Pfs16* Tajima’s D values against region - see supplementary data sheet 2). However, *An. funestus* vector occurrence (as a binomial variable) had a significant relationship with region (analysis of variance p value = 0.0493). In addition to the regression analyses mentioned above, we created a violin plot of the Pfs47 haplotype diversity, Pfs16 haplotype diversity and Pfs16 Tajima’s D values for different vector species combinations. In those plots, we observed that higher (i.e. less negative) Tajima’s D values and haplotype diversities at Pfs16 were associated with the combinations that included *An. funestus* (Supplementary Fig. 5, Supplementary data sheet – ANOVA sheet).

### 3.5 Comparative analysis of P47Rec in *An. gambiae* and *An. stephensi*

**Figure 5:**
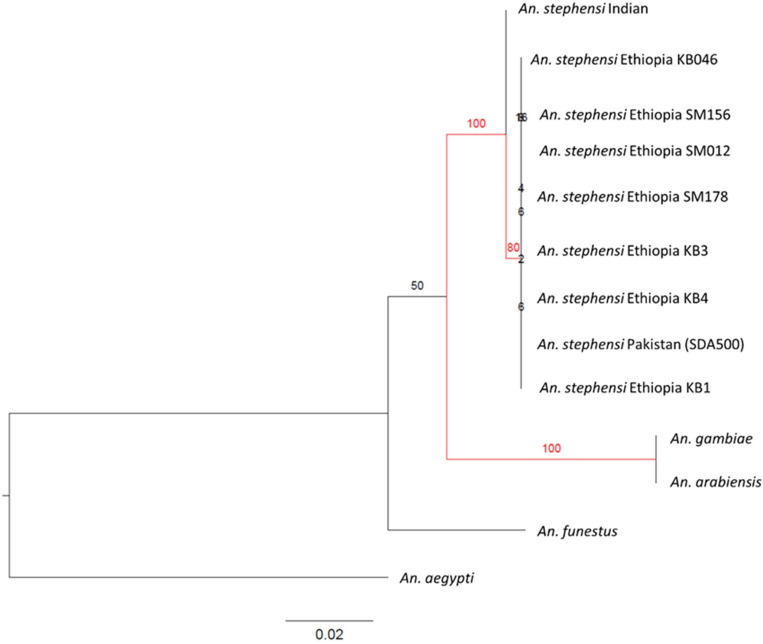
Maximum likelihood tree for the P47Rec orthologs of prominent malaria vectors in Africa with P47Rec ortholog of *An. stephensi* collected in Ethiopia (500 bootstraps). The significant branches with bootstrap values higher than 70 are highlighted in red.

In *An. gambiae*, the P47Rec amino acid sequence was very well conserved within the sequences we observed in Ethiopia. With the invasion of *An. stephensi* into the Horn of Africa (HOA) we wanted to investigate the differences in amino acid sequence of P47Rec ortholog in *An. stephensi* that could have an impact on compatibility between invasive vector and existing *Plasmodium* populations in the HOA. There are twenty-eight amino acid changes among all the prominent malaria vectors in Africa (*An. funestus, An. melas, quadriannulatus, An. arabiensis, An. merus* and *An. gambiae*) in P47Rec orthologs reported by Molina-cruz et. al in 2020 (16). There were eighteen amino acid changes (listed in Table 3) observed in P47Rec amino acid sequences between *An. stephensi* collected in Ethiopia and *An. gambiae* reference sequence compared to twenty-five observed between *An. gambiae* and *An. funestus*. Out of the eighteen, thirteen amino acid differences overlapped with the changes observed among all prominent African vectors and five were unique to *An. stephensi*. No differentiation was observed between *An. stephensi* from Kebridehar and Semera. Further, we performed phylogenetic analysis of the coding sequence of the *P47Rec* gene (Fig. 5) including *An. gambiae sl*, *An. funestus*, *An. arabiensis*, and *An. stephensi* (sequences from Indian strain, Pakistani strain, and Ethiopia). The results indicated that haplotype of the P47Rec ortholog in *An. stephensi* from Ethiopia is closer to the *An. stephensi* from Pakistan (SDA500) compared to the Indian strain (separation was supported by bootstrap value = 80).

## 4. DISCUSSION

### 4.1 Limited evidence of vector-mediated selective pressure on P. falciparum populations through P47 system within Africa.

The P47 system was the best starting point to investigate vector-mediated selective pressure on parasite variation on a subcontinental scale because of the well-established molecular interaction of the *P. falciparum* Pfs47 and *An. gambiae* P47Rec proteins and their role in parasite invasion of mosquito midgut. In this study, we observed many *Pfs47* haplotypes overall but no correlation between haplotype distribution and geography. Also, no evidence of selection was detected according to the Tajima’s D values observed in the parasite populations studied here. Furthermore, the P47Rec amino acid sequence is highly conserved among *Anopheles gambiae* populations, indicating the absence of vector subpopulation variation necessary to drive selection by *An. gambiae* alone. Even though we see *Pfs47* haplotype diversity within each population, the pattern of diversity in *Pfs47* suggests only neutral processes at play. This does not necessarily contradict previous studies that show evidence of differing *P. falciparum* haplotype compatibility across continentally structured *Anopheles* species. (9). While there are divergent *Anopheles* species that exist within Africa, the portions of the *P47Rec* that drive compatibility may be conserved in African *Anopheles*, leading to less restrictions on transmission specificity and the maintenance of diversity in *Pfs47*. Further functional analysis of *P47Rec* is needed to evaluate the precise genic regions at play in compatibility.

Interestingly, we also detected a new mutation (M219I) not yet reported in central domain of the *Pfs47* Del region which has been chosen to use as an antigen for TBV for malaria. Thus, these findings have implications for the eficacy of vaccines, given that the native vectors can transmit this strain with these variants.

### 4.2 Diversity in *An. gambiae* genes HPX15 and APN1

In contrast to P47Rec, both HPX15 and APN1 were highly diverse on the amino acid level. Phylogenetic analysis supported multiple distinct groups for HPX15 (bootstrap values > 70) but not for APN1 (bootstrap values < 70). The observed sub-species differentiation in HPX15 indicates the potential for this gene to serve as a driver of selection on its matching *P. falciparum* gene in the parasite. Further investigation of this locus coupled with the identification of their corresponding *P. falciparum* surface proteins will elucidate whether vector-population-mediated selective pressure is occurring. We anticipate detecting signatures of balancing selection in the HPX15 ligand(s) protein within *P. falciparum* populations across Africa due to the subpopulation variation observed at this locus on *An. gambiae*.

### 4.3 Potential signals in *P. falciparum* Pfs16

While the study of the P47 system revealed limited evidence of ongoing vector-population-mediated selection on the *Pfs47* within Africa, differing patterns of diversity were observed at *Pfs16*. Fewer haplotypes were observed at this location with no geographic structure (protein haplotypes not with nucleotide haplotypes). Furthermore, a signal of positive selection or population expansion was observed in each study population at *Pfs16.* After comparing the Pfs16 Tajima’s D values with genome-wide values we could rule out the possibility of observing negative Tajima’s D values due to population expansion. This indicates that selection has occurred more recently at this locus within central Africa. We also observed two important mutations, a single non-synonymous mutation and a 9 nucleotide (3 amino acid) indel. While the receptor for Pfs16 in vectors has not been identified yet, these findings support further investigation of this gene and potential receptors in the vector.

### 4.4 Association analysis supports vector-mediated selection

Nucleotide diversity and haplotype diversity in genes crucial for vector-parasite interactions in *P. falciparum* in Africa could be significantly influenced by the composition of vector species in malaria-endemic regions. However, we could not find a way to obtain vector composition data for all the locations where the *P. falciparum* samples were collected for this study. To test the above hypothesis, we downloaded the predicted vector occurrence probability values of the prominent malaria vectors for the locations of parasite samples were collected, from Malaria Atlas Project (MAP) and investigated the relationships between haplotype diversities and Tajima’s D values of the genes important for the interaction with vectors in *P. falciparum* in Africa (39, 40). We observed a statistically significant correlation between amino acid haplotype diversities of parasite genes (Pfs47 and Pfs16) and predicted occurrence probabilities of vector species *An. arabiensis*, *An. funestus and An. moucheti* individually. In addition to the individual associations, the above observation was confirmed by the association between higher haplotype diversities at Pfs16 and combined occurrence of *An. arabiensis* and *An. funestus* compared to the occurrence of a single vector. These signals could be an indication of vector-mediated selection on parasite populations. However, we cannot rule out the impact of geography-correlated variables (eg. transmission intensity, ancient genetic variation clines) on diversity due to the significant p-values observed in one-way ANOVA between amino acid haplotype diversity and the region of the African continent (Central, East and West).

To investigate the impact of VOP on selection more directly, we examined the relationship between Tajima’s D values of *Pfs16* (*Pfs16* was the only gene with significant Tajima’s D values) and VOP of mosquito species that had a statistically significant correlation with parasite gene haplotype diversities individually. We found an association between *An. funestus* and *Pfs16* Tajima’s D values, such that the presence of *An. funestus* was associated with a lower signal of positive selection. Since *An. funestus*, genetically distinct from *An. arabiensis*, and *An. moucheti* which are both part of the *An. gambiae* complex, it is possible to observe a lower signal of positive selection due to the presence of multiple genetically distinct vector haplotypes to facilitate the transmission of *P. falciparum.* A limitation in this analysis is the availability of data on the lower end of *An. funestus* occurrence probability distribution. We have a single datapoint which is coming from Kilifi in Kenya (the only datapoint from Kenya). According to both Malaria Atlas Project prediction of 2010 (39) and 2017 (40), Kenya has a low occurrence probability of *An. funestus*. It is also important to mention that the vector occurrence probabilities in Malaria Atlas Project were predicted values based on the geographical and environmental factors, which could add more ambiguity/noise to the estimate values of correlation analysis and regression analysis. Ultimately, these results indicate that there could be an influence from the vector species composition on the selection of the parasite genes important for the interaction with vectors.

### 4.5 Implications for *An. stephensi* invasion in Africa

Given that *Pfs47* in African *P. falciparum* populations exhibited signals of neutral evolution in relation to the current sympatric vector populations (*An. gambiae*, *An. funestus*, etc.), we aimed to investigate how the evolution of *Pfs47* might be influenced by the introduction of *An. stephensi*. As an initial step, we examined the *P47rec* ortholog in *An. stephensi*. Since the P47Rec coding sequence in *An. gambiae* in Africa was fully conserved, we wanted to investigate the number of amino acid changes in P47Rec ortholog in invasive *An. stephensi*. We compared the amino acid sequence of the P47Rec ortholog in *An. stephensi* in Ethiopia to the *An. gambiae* gene and found eighteen amino acid differences. These findings combined with phylogenetic analysis indicating differentiation between *An. stephens*i and African species (*An. gambiae* bootstrap value = 100 and *An. funestus* bootstrap value = 100) support the potential for new *P. falciparum* haplotype compatibilities in Africa with the arrival, spread, and establishment of invasive *An. stephensi*. In addition, the phylogenetic analysis revealed a close relationship between P47Rec in invasive *An. stephensi* and the SDA500 *An. stephensi* strain (bootstrap = 100). The SDA500 strain is known to be highly susceptible to both I248L haplotypes in Pfs47 in *P. falciparum* (18). Therefore, the presence of similar P47Rec sequences in the invasive *An. stephensi* supports predictions of the gradual emergence of new *Pfs47* haplotypes in Africa.

### 4.6 Future Directions

In this study we focused on several genes known to be important for vector-parasite interactions of malaria and their role in shaping the population structure of *P. falciparum* parasites through selective forces exerted from vector populations. There could be many other genomic factors that can influence the vector-parasite interactions and studies should include investigation of additional genes. This study did not include samples from all the malaria endemic countries in Africa. In addition, the samples were collected in multiple years that expanded over two decades. In a future study we expect to broaden our list of genes used in the analyses and to include samples from other malaria endemic countries in Africa.

## 5. Conclusion

This study provides preliminary insight into the potential for sub-vector species level and multiple vector species selective pressure impacting *Plasmodium*-*Anopheles* compatibility within Africa. Notably, these findings support the notion that compatibility is complex and in addition functional, and population genetic investigations are needed. Furthermore, this study provides the first analysis to explore how occurrence of multiple vectors and invasive *An. stephensi* could change parasite diversity in multiple African countries. Finally, the current structure of diversity revealed at these transmission relevant loci have major implications for the design and eficacy of vaccines and antimalarial treatments in *An. stephensi* invaded regions.

## Supplementary Material

Supplement 1

## Figures and Tables

**Table 1:** The number of non-synonymous mutations (NSMs) per exon in *An. gambiae* genes

Gene	Exon	# of NSMs	# of NSM with freq. > 0.01
P47Rec	1	0	0
	2	2	0
	3	2	0
	4	5	0
	**Total**	**9**	**0**
HPX15	1	27	5
	2	44	17
	3	38	10
	**Total**	**109**	**32**
APN1	1	23	7
	2	18	10
	3	4	0
	4	25	3
	5	60	52
	**Total**	**130**	**72**

**Table 2:** Observed significant Kendall’s rank correlation tau estimates and p-values for the amino acid haplotype diversities of parasite genes and predicted vector occurrence probabilities based on 2010 prediction model.

		An. arabinesis	An. funestus	An. moucheti
Pfs47	tau estimate	−0.4217757	−0.3807641	0.434292
	p-value	0.01082	0.02068	0.02132
Pfs16	tau estimate	0.6099269	0.5013643	−.4863484
	p-value	0.0002694	0.002899	0.01087
Pfs37	NA	Non-significant	Non-significant	Non-significant

**Table 3:** The amino acid differences between *An. gambiae* and *An. stephensi* coding sequences of P47Rec.

Exon	Amino acid change (from *gambiae* to *stephensi*)
Exon 2	G23A~
	I34V~
	Q53H*
	T67S*
Exon 3	S81G~
	N90G~
	I105V~
	I119V*
	G131N~
Exon 4	V165I~
	N169H~
	G173S~
	T181A~
	S195T
	T217V~
	Q220N~
	S242T*
	A252T*

* The amino acid changes marked with are changes unique to *An. gambiae* and *An. stephensi* collected in Ethiopia and amino acid changes marked with ~ are observed among the other prominent malaria vectors (*An. funestus, An. melas, quadriannulatus, An. arabiensis, An. merus*) and *An. gambiae*.

## Data Availability

All data generated or analyzed during this study are included in this published article and its supplementary information files. The *P. falciparum* and *An. gambiae* genome datasets used in this study were obtained from publicly available databases, including the MalariaGen PF6K and Ag1000 datasets, respectively, in accordance with their data-sharing policies. The P47Rec ortholog sequences extracted from An. stephensi mosquito collected in Ethiopia can be found in the supplementary data file – “all_P47Rec_CDS.fas” with supplementary data.
