# Supplementary material for: Multi-locus investigation of Anopheles-mediated selective pressure on Plasmodium falciparum in Africa
Source: Res Sq. 2024 Oct 30:rs.3.rs-5040478. Preprint. [Version 1] doi: 10.21203/rs.3.rs-5040478/v1 (PMC11581053; doi:10.21203/rs.3.rs-5040478/v1)
Supplement: Supplement 1 [file NIHPPRS5040478V1-supplement-1.pdf]

Supplementary Files

This is a list of supplementary files associated with this preprint. Click to download.

- Supplementaryfigure1.png
- Supplementaryfigure2.png

- Supplementaryfigure3.png
- Supplementaryfigure4.png
- Supplementaryfigure5.png
- Supplementaryfigure61000BSAPN1fullCDSAAtree.pdf
- Supplementaryfigure71000BSHPX15fullCDSAATree.pdf
- supplementarytable1P.falciparumSampleDetails.csv
- supplementarytable2An.gambiaeSampleDetails.csv
- Supplementarydatasheet2linearregressionsandANOVAs.xlsx
- allP47RecCDS.fas
